# The disease-causing tau V337M mutation induces tau hypophosphorylation and perturbs axon morphology pathways

**DOI:** 10.1101/2024.06.04.597496

**Published:** 2024-06-06

**Authors:** Gregory A. Mohl, Gary Dixon, Emily Marzette, Justin McKetney, Avi J. Samelson, Carlota Pereda Serras, Julianne Jin, Andrew Li, Steven C. Boggess, Danielle L. Swaney, Martin Kampmann

**Affiliations:** 1. Institute for Neurodegenerative Diseases, University of California San Francisco, San Francisco, CA, USA; 2. Department of Neurology, University of California San Francisco, San Francisco, CA, USA; 3. Gladstone Data Science and Biotechnology Institute, The J. David Gladstone Institutes, San Francisco, CA, USA; 4. Quantitative Bioscience Institute, University of California San Francisco, San Francisco, CA, USA; 5. Department of Cellular and Molecular Pharmacology, University of California San Francisco, San Francisco, CA, USA; 6. Bakar Computational Health Sciences Institute, University of California San Francisco, San Francisco, CA USA; 7. Department of Biochemistry and Biophysics, University of California San Francisco, San Francisco, CA, USA

## Abstract

Tau aggregation is a hallmark of several neurodegenerative diseases, including Alzheimer’s disease and frontotemporal dementia. There are disease-causing variants of the tau-encoding gene, *MAPT*, and the presence of tau aggregates is highly correlated with disease progression. However, the molecular mechanisms linking pathological tau to neuronal dysfunction are not well understood due to our incomplete understanding of the normal functions of tau in development and aging and how these processes change in the context of causal disease variants of tau. To address these questions in an unbiased manner, we conducted multi-omic characterization of iPSC-derived neurons harboring the *MAPT* V337M mutation. RNA-seq and phosphoproteomics revealed that both V337M tau and tau knockdown consistently perturbed levels of transcripts and phosphorylation of proteins related to axonogenesis or axon morphology. Surprisingly, we found that neurons with V337M tau had much lower tau phosphorylation than neurons with WT tau. We conducted functional genomics screens to uncover regulators of tau phosphorylation in neurons and found that factors involved in axonogenesis modified tau phosphorylation in both *MAPT* WT and *MAPT* V337M neurons. Intriguingly, the p38 MAPK pathway specifically modified tau phosphorylation in *MAPT* V337M neurons. We propose that V337M tau might perturb axon morphology pathways and tau hypophosphorylation via a “loss of function” mechanism, which could contribute to previously reported cognitive changes in preclinical *MAPT* gene carriers.

## Introduction

Neurodegenerative diseases are a growing public health burden and remain very challenging to treat because we lack a complete understanding of the underlying disease mechanisms. A common theme in many neurodegenerative diseases is the aggregation of pathological proteins [1]. Tau aggregation is a hallmark of neurodegenerative diseases collectively called tauopathies, including Alzheimer’s disease and frontotemporal dementia. In Alzheimer’s disease, tau aggregation and phosphorylation changes correlate better with disease progression than amyloid beta pathology [2] despite clear genetic evidence linking amyloid beta to the disease [3], suggesting a complex process in which amyloid beta, pathological tau, immune activation, and synapse loss likely collaborate to drive disease. In frontotemporal dementia, rare causal variants of tau that are fully penetrant for the disease prove a direct role for tau in disease pathogenesis [4].

Despite the tremendous progress made in the field, there are many unanswered questions about how pathogenic tau causes disease. Recent work in human iPSC-derived neurons has shown that pathogenic variants of tau sensitize neurons to many different types of cellular stress and that this effect can be rescued by lowering tau levels via autophagy [5]. Other groups have shown that tau interferes with RNA splicing and stress granules homeostasis [6–9], disrupts the nuclear envelope [10–12], perturbs axonal trafficking [13, 14] or disrupts mitochondrial dynamics [15]. Acetylated tau has also been shown to disrupt chaperone mediated autophagy, rerouting tau and other clients to be degraded by other mechanisms [16]. Pathogenic tau has also been shown to perturb plasticity of the axon initial segment and cause changes to neuronal excitability [17] and has been implicated in driving excitotoxicity [9, 18–20]. Many of these data support a tau toxic gain-of-function model, and tau lowering has been successfully shown to be beneficial in cultured neurons and animal models [5, 21]. In fact, tau lowering is currently being tested in the clinic by antibodies and ASOs, and tau lowering is one of the most promising approaches for these devastating diseases [22]. It is clear from this broad body of work over the last 20 years that tau can induce many cellular phenotypes under different conditions and in different contexts.

To characterize the earliest changes that pathogenic tau causes in human neurons and to understand mechanistically how pathogenic tau causes human disease, we used a multi-omic approach to unbiasedly determine the cellular phenotypes linked to pathogenic tau. This is essential because many focused studies have linked tau to diverse cellular processes that go awry in neurodegeneration, but there are few unbiased or comprehensive studies. We modeled pathogenic tau by using human iPSC-derived neurons with the *MAPT* V337M mutation, a known cause of frontotemporal dementia. We used two sets of induced pluripotent stem cells (iPSCs), one from a healthy donor (WTC11) and one from a patient with the *MAPT* V337M mutation (GIH6C1) [17, 23].

Our RNA-seq, ATAC-seq, proteomics and phosphoproteomics results all point to changes in axonogenesis due to the *MAPT* V337M mutation. Recently published mouse phosphoproteomics datasets in tau knockout mice and P301S mice strongly support the link between tau and axonogenesis factors and intriguingly suggest that these effects are due to tau loss of function [24, 25]. Tau knockdown in *MAPT* WT and *MAPT* V337M neurons showed that changes in axonogenesis regulation are phenocopied by tau knockdown in *MAPT* WT neurons and are not rescued by tau knockdown in *MAPT* V337M neurons. *MAPT* V337M neurons have hypophosphorylated tau, which is recapitulated by artificially overexpressing V337M tau but not WT or R406W tau in neurons with endogenous tau knockdown. Unbiased CRISPR screens for regulators of tau phosphorylation uncovered axonogenesis-related regulators of tau phosphorylation and show that the p38 MAPK pathway may play a role in modifying tau phosphorylation specifically in V337M neurons. We propose that V337M tau might perturb axon morphology pathways and tau phosphorylation via a “loss-of-function” mechanism, which could contribute to previously described cognitive changes in preclinical *MAPT* gene carriers.

## Results

### MAPT V337M and MAPT knockdown perturb the transcription of axonogenesis-related genes

**Figure 1:**
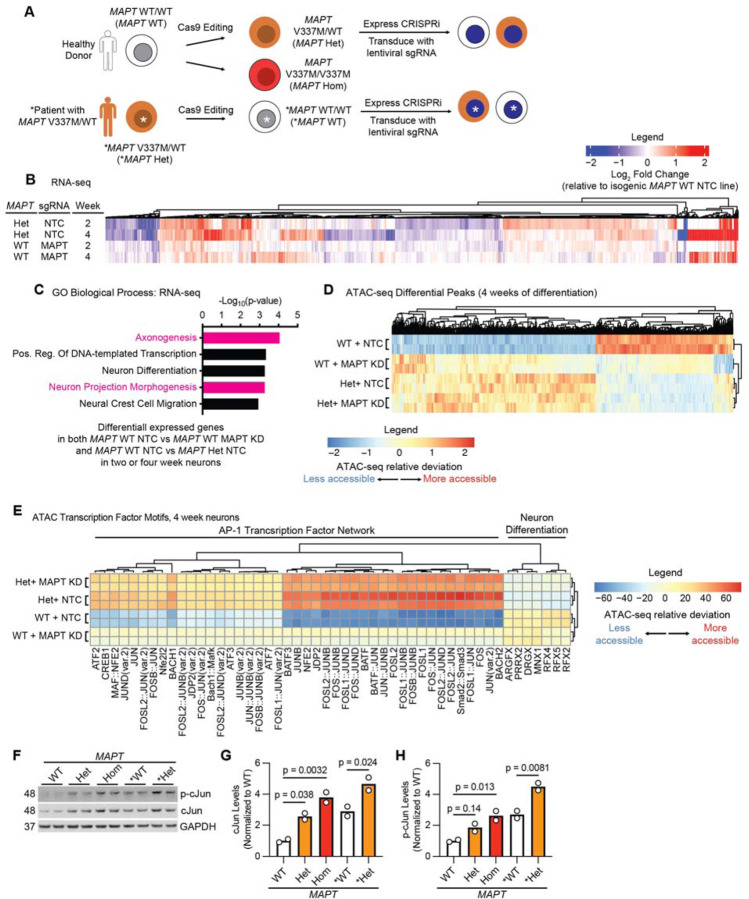
RNA-seq and ATAC-seq in neurons reveal conserved effects of the *MAPT* V337M mutation on in axonogenesis that mirror tau reduction. **(A) (***Top Left*, iPSCs from a healthy donor (WTC11, here called *MAPT* WT) were edited with Cas9 in previous work to generate a heterozygous *MAPT* V337M clone (*MAPT* Het) and a homozygous *MAPT* V337M clone (*MAPT* Hom). *Bottom Left,* iPSCs generated from a patient with the heterozygous *MAPT* V337M mutation (GIH6C1, here called *MAPT Het) were edited with Cas9 in previous work to generate a healthy isogenic control (GIH6C1∆1E11, here called *MAPT WT). These cells were engineered to express a dox-inducible mNGN2 in the AAVS1 safe harbor locus and CRISPRi machinery in the CLYBL locus. We then transduce the iPSCs with lentivirus for sgRNA and BFP expression. **(B)** Heatmap comparing changes in gene expression based on RNA-seq in *MAPT* Het NTC and *MAPT* WT MAPT KD vs. *MAPT* WT NTC at 2 and 4 weeks post differentiation. Three replicates (independent wells) of neurons for each genotype/sgRNA combination were harvested at each timepoint. **(C)** Gene Ontology (GO) term enrichment analysis of the RNA-seq experiment in (B). Genes that are differentially expressed in both *MAPT* Het and *MAPT* WT *MAPT* KD vs. *MAPT* WT NTC were analyzed with Enrichr, and top terms with minimal overlap were plotted. Pathways related to axonogenesis and neuron morphology are colored magenta. **(D)** Heatmap summarizing ATAC-seq differential peaks at 4 weeks of differentiation. Two replicates (independent wells) of neurons for each genotype/sgRNA combination were harvested. ATAC-seq relative deviation is represented, with blue or negative values reflecting less accessible chromatin and red or positive values reflecting more accessible chromatin. **(E)** Heatmap summarizing ATAC-seq transcription factor motif analysis at 4 weeks of differentiation derived from the same experiment shown in (D). ATAC-seq relative deviation is represented, with blue or negative values reflecting less accessible chromatin and red or positive values reflecting more accessible chromatin for the transcription factor motifs across the genome. Clusters were analyzed for pathway enrichment using Enrichr, and major pathways are annotated (“AP-1 Transcription Factor Network” and “Neuron Differentiation”). **(F)** Western blot measuring p-cJun and cJun levels in neurons at 1 week of differentiation. Two replicates (independent wells) of neurons for each genotype/sgRNA combination were harvested. **(G-H)** Quantification of cJun (G) and p-cJun (H) from the western blot in (F). Bars represent the mean of 2 data points. Significance was calculated using one-way ANOVA with Dunnett’s multiple comparison test, and comparisons were restricted within the donor background.

iPSCs generated from a healthy individual (WTC11, referred to as *MAPT* WT [26]) or an FTD patient with the *MAPT* V337M mutation (GIH6C1, referred to as *MAPT Het [23]) were edited in previous work [17, 23] with Cas9 to generate isogenic pairs either introducing or correcting the *MAPT* V337M mutation (Fig. 1A). The *MAPT* WT iPSCs were edited with Cas9 to generate a heterozygous *MAPT* V337M/WT clone (*MAPT* Het) and homozygous *MAPT* V337M/V337M clone (*MAPT* Hom). The *MAPT Het iPSCs were corrected with Cas9 to generate a *MAPT* WT/WT clone (*MAPT WT). We engineered the GIH6C1 lines to introduce a doxycycline-inducible Ngn2 for neuronal differentiation and CRISPRi machinery. We transduced the iPSCs with lentiviral sgRNAs targeting *MAPT* to knock down tau or non-targeting control (NTC) sgRNAs for further mechanistic characterization (Figure S1A–C).

RNA-seq of neurons harvested at 2 and 4 weeks of differentiation revealed overlap between effects in *MAPT* Het neurons and *MAPT* WT tau knockdown neurons (Figure 1B). Genes that were differentially expressed in *MAPT* Het neurons and *MAPT* WT tau knockdown neurons were significantly enriched for regulators of axonogenesis (Figure 1C). Knocking down tau in *MAPT* Het neurons resulted in only five differentially expressed genes (Figure S1D). Differentially expressed genes in *MAPT* Hom and *MAPT Het neurons compared to isogenic controls were also significantly enriched for regulators of axonogenesis, even at one week of differentiation (Figure S1E–G). ATAC-seq at 2 and 4 weeks of differentiation showed similar patterns as the RNA-seq (Figure 1D, Figure S2A), and genes with differentially accessible peaks proximal to their transcription start site (TSS) were enriched for axon-related genes (Figure S2B). Transcription factor motif analysis showed that motifs for the AP-1 Transcription factor network, which includes the cJun family of transcription factors, were consistently more accessible in *MAPT* Het and *MAPT* WT tau knockdown neurons compared to controls (Figure 1E, Figure S2C). We hypothesized that cJun phosphorylation may be changed in *MAPT* V337M neurons since cJun activity is known to be regulated by phosphorylation. Indeed, both p-cJun and cJun levels were increased in *MAPT* Het, *MAPT* Hom and *MAPT Het neurons vs. isogenic controls (Figure 1F–H). *MAPT* V337M and tau knockdown induce changes in chromatin accessibility and transcription of axonogenesis-related genes with surprising overlap, suggesting that some phenotypes in *MAPT* V337M neurons may be due to a loss of normal tau function, rather than a gain in toxic function.

### MAPT V337M and tau knockdown perturb phosphorylation of axonogenesis-related proteins

**Figure 2:**
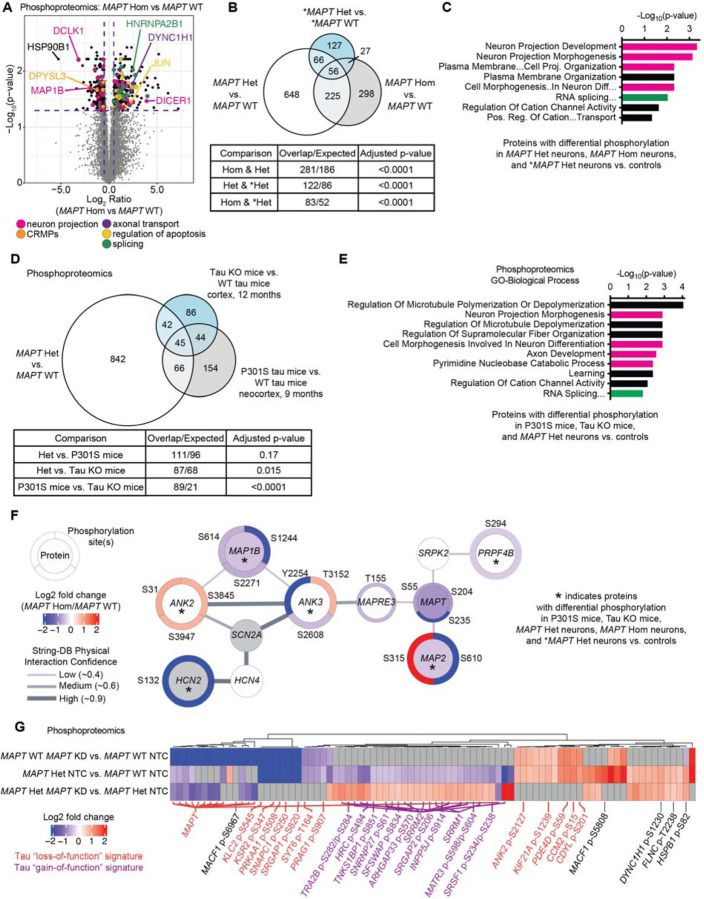
Proteomics shows convergence on altered phosphorylation of axonogenesis-related proteins in neurons with the *MAPT* V337M mutation. **(A)** The effect of *MAPT* V337M on proteome phosphorylation in neurons was quantified using mass spectrometry. Four replicates (independent 150mm dishes) of neurons for each genotype/sgRNA combination were harvested after one week of differentiation. Volcano plot showing changes in protein phosphorylation in *MAPT* V337M homozygous (*MAPT* Hom) vs. *MAPT* WT neurons. Proteins are color-coded by pathways based on GO annotations. Dots represent individual phosphorylation sites. **(B)** Overlap of proteins with differential phosphorylation between *MAPT* V337M heterozygous neurons (*MAPT* Het), *MAPT* Hom neurons, and *MAPT* V337M heterozygous neurons derived from patient iPSCs (*MAPT Het) vs. isogenic controls. Significance was calculated using multiple t-tests adjusted with Šidák single-step correction. Proteins with significantly different phosphorylation sites in all three datasets were filtered to identify 56 conserved proteins. **(C)** GO term enrichment of the 56 proteins with differential phosphorylation between *MAPT* Het neurons, *MAPT* Hom neurons, and *MAPT Het neurons vs. isogenic controls. Bars for terms related to axonogenesis or neuron morphology are colored in magenta, and bars for splicing terms are colored in green. The term “Plasma Membrane…Cell Proj. Organization” is abbreviated from “Plasma Membrane Bounded Cell Projection Organization.” The term “Cell Morphogenesis…In Neuron Diff…” is abbreviated from “Cell Morphogenesis Involved In Neuron Differentiation.” The term “RNA splicing…” is abbreviated from “RNA Splicing, Via Transesterification Reactions With Bulged Adenosine As Nucleophile.” The term “Pos. Reg. of Cation…Transport” is abbreviated from “Positive regulation of Cation Transmembrane Transport.” **(D)** Overlap of proteins with differential phosphorylation between *MAPT* Het vs. *MAPT* WT and two published mouse phosphoproteomics datasets, one from tau KO mice and another from P301S mice compared to WT mice. Significance was calculated using multiple t-tests adjusted with Šidák single-step correction. Proteins with significantly different phosphorylation sites in all three datasets were filtered to identify 45 conserved proteins. **(E)** GO term enrichment of the 45 proteins with differential phosphorylation between *MAPT* Het neurons, tau KO mice and P301S mice vs. controls. Bars for terms related to axonogenesis or neuron morphology are colored in magenta, and bars for splicing terms are colored in green. The term “RNA Splicing…” is abbreviated from “RNA Splicing, Via Transesterification Reactions With Bulged Adenosine As Nucleophile.” **(F)** String-DB protein-protein interaction network of proteins with differential phosphorylation in five datasets: *MAPT* Het, *MAPT* Hom, *MAPT Het vs isogenic controls, and tau KO mice and P301S mice vs. controls. Six proteins were identified in all five datasets and are marked by larger circles, including *HCN2*, *ANK2*, *ANK3*, *MAP1B*, *MAP2*, and *PRP4FB*. *MAPT*, *SRPK2*, *MAPRE3*, *SCN2A*, and *HCN4* were included as part of the shortest path to connecting the network. The inner circle is colored based on the protein log_2_ fold change in *MAPT* Hom vs *MAPT* WT, and the outer circles are colored based on the log_2_ fold change for the indicated phosphorylation site in *MAPT* Hom vs. *MAPT* WT. Significant proteins and phosphorylation sites are labeled in black text, and non-significant proteins are labeled in grey text. The thickness of the connecting lines represents the confidence of the protein-protein interaction based on the String-DB database. **(G)** Heatmap of phosphoproteomics data comparing *MAPT* KD in *MAPT* WT and *MAPT* Het neurons vs. isogenic controls. Phosphosites that are decreased in *MAPT* Het vs. *MAPT* WT but that are rescued by tau knockdown in *MAPT* Het neurons are labeled in purple as a potential tau “gain of function” signature. Phosphosites that are changed in the same direction in *MAPT* WT *MAPT* KD and *MAPT* Het vs. WT are labeled in orange as a potential tau loss of function signature. GO term analysis for these proteins are in Supplemental Figure 2H-I.

We hypothesized that changes in p-cJun may reflect broad changes in cell signaling caused by V337M tau. To address this question, we determined the total proteome and phosphoproteome of neurons with *MAPT* V337M and/or tau knockdown by mass spectrometry. Phosphoproteomic analysis of *MAPT* V337M neurons confirmed elevated p-cJun levels while also uncovering differential phosphorylation for proteins regulating neuron projection development and splicing (Figure 2A,C). There was significant overlap in the proteins with differential phosphorylation between *MAPT* Hom, *MAPT* Het and *MAPT Het neurons vs. isogenic controls (Figure 2B), though the identities of the differential phosphosites varied between conditions (Figure S3A). Gene set enrichment analysis for the 56 conserved proteins with changes in phosphorylation in *MAPT* V337M neurons showed that the top enriched terms were related to neuron projection development (Figure 2C). The total protein levels for many of these factors were not significantly changed, suggesting that these changes are due to specific signaling events altering phosphorylation patterns, rather than just changes in protein levels (Figure S3B–D).

We next compared our phosphoproteomic datasets to recently published mouse phosphoproteomic datasets using tau knockout mice [24] or P301S tau mice [25]. We found significant overlap for proteins with differential phosphorylation in our data and the tau knockout mice but not with the P301S mice (Figure 2D). However, we also noted that there was significant overlap between the tau knockout mice and the P301S mice. Gene set enrichment analysis identified substantial enrichment of axonogenesis-related protein phosphorylation changes in the 45 conserved proteins with differential phosphorylation in *MAPT* V337M neurons, tau knockout mice, and P301S tau mice (Figure 2E). When we determined an even more focused set of proteins that also have differential phosphorylation in *MAPT* V337M homozygous and *MAPT* V337M heterozygous neurons from patient iPSCs, we found a core network of proteins in highly related pathways regulating neuron morphogenesis and polarity (Figure 2F), including *ANK3* and *MAPRE3*. *ANK3* and *MAPRE3* were recently identified to be important for V337M tau-induced defects in axon initial segment plasticity [17].

We observed two patterns of protein phosphorylation changes due to tau knockdown (Figure 2G). Many phosphorylation changes were specific to either tau knockdown in the *MAPT* WT neurons or the *MAPT* V337M neurons. When we performed gene set enrichment analysis on proteins with differential phosphorylation in *MAPT* WT tau knockdown neurons, the only significantly enriched term was “Regulation of microtubule-based process,” with many of these proteins being involved in axonogenesis (Figure S3E). Gene set enrichment analysis of proteins with differential phosphorylation in *MAPT* V337M tau knockdown showed that splicing factors were predominantly affected, whereas cytoskeletal and axonogenesis proteins were not perturbed (Figure S3F).

### V337M tau is hypophosphorylated in neurons

**Figure 3:**
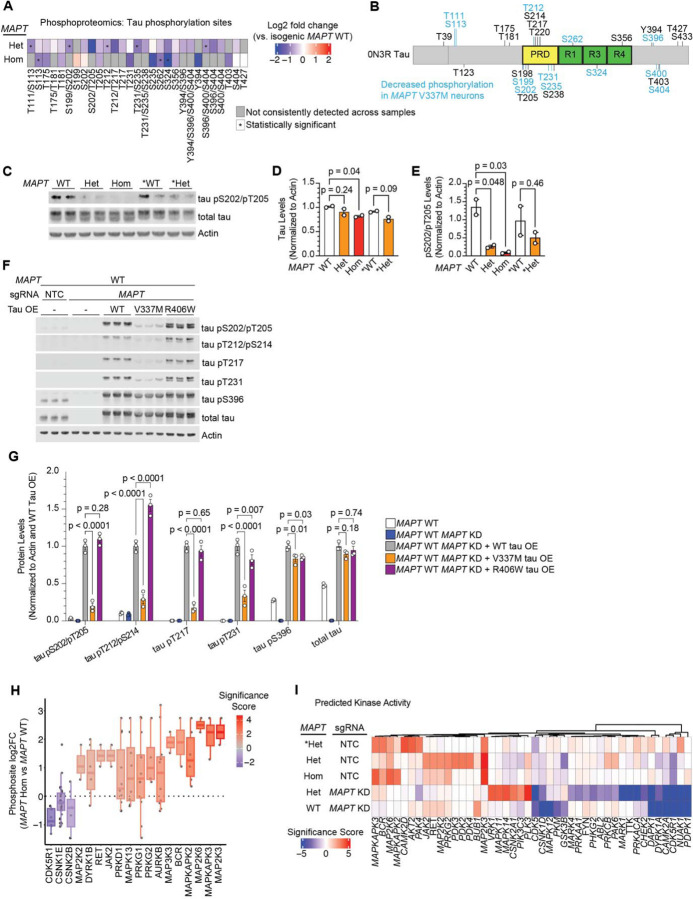
Tau phosphorylation is reduced in neurons with the *MAPT* V337M mutation. **(A)** Heatmap of tau phosphorylation from *MAPT* V337M neurons with a homozygous *(MAPT* Hom) or heterozygous mutation *(MAPT* Het) compared to isogenic controls *(MAPT* WT).Tau was not detected in patient-derived *MAPT V337M heterozygous neurons (*MAPT Het) vs. isogenic controls (*MAPT WT) due to poor coverage in these samples. Phosphosites that were not detected in more than half of the replicates in both samples are marked in grey, and statistically significant phosphorylation changes are marked with an asterisk. **(B)** Protein domain map of 0N3R tau with detected phosphorylation sites labeled. Decreased phosphorylations detected in either *MAPT* Hom or *MAPT* Het neurons are labeled in blue. In cases where the phosphoproteomics could not distinguish between multiple potential phosphosites, all are included. Abbreviations for domains are as follows: proline-rich domain (PRD), microtubule binding repeat domains (R1, R3, R4). The location of the N-terminal N1/N2 domains that are excluded from 0N3R tau are marked by a grey line. **(C)** Western blot validating decreased tau phosphorylation in neurons with V337M tau. Two replicates (independent wells) of neurons were harvested after one week of differentiation. AT8 was used to label tau pS202/pT205, and Tau13 was used to label total tau. **(D)** Quantification of total tau levels from the western blot in (E). One way ANOVA with Šidák’s correction and comparisons within donor backgrounds was used to test for significance. **(E)** Quantification of tau pS202/pT205 levels from the western blot in (E). One way ANOVA with Šidák’s correction and comparisons within donor backgrounds was used to test for significance. **(F)** WT, V337M and R406W tau were overexpressed via lentivirus in *MAPT* WT *MAPT* KD iPSCs. Three replicates (independent wells) of neurons were harvested after one week of differentiation. pTau and total tau levels were analyzed by Western blot in the control and overexpression lines. **(G)** Quantification of the western blot in (F). pTau and Tau band intensity was normalized to actin and then to the WT tau overexpression line. Significance was calculated using two-way ANOVA with Dunnet’s multiple comaprisons test. **(H)** Kinase activity analysis from phosphorylation changes in neurons after one week of differentiation with the homozygous *MAPT* V337M mutation (*MAPT* Hom) vs. isogenic controls (*MAPT* WT). The log_2_ fold change of phosphopeptide abundance for annotated kinase substrates is plotted. The significance is calculated based on the number of known substrates and how many substrates are modified in the same direction. The range is represented by the thin lines, the box represents the IQR, and the median is represented by a thick line. **(I)** Heatmap for kinase activity scores from all five phosphoproteomic datasets vs. isogenic controls.

We observed that *MAPT* V337M neurons had lower tau phosphorylation occupancy as compared to WT across all domains of the protein at many sites (Figure 3A and 3B) and validated these changes by western blot in all sets of neurons (Figure 3C–E). Many of the differential phosphorylation sites are known to be hyperphosphorylated in Alzheimer’s disease and other tauopathies [27–29] (Figure S4A).

To further explore how V337M tau may have decreased phosphorylation in neurons, we overexpressed WT tau, V337M tau or R406W tau in *MAPT* WT neurons with endogenous tau knocked down. Consistent with our phosphoproteomics results, V337M tau had decreased phosphorylation at numerous sites despite having similar tau levels to WT tau and R406W tau (Figure 3F,G). Intriguingly, R406W tau did not have decreased phosphorylation at most of these sites. These data suggest tau variants affect tau phosphorylation in neurons via distinct mechanisms.

Extensive work has been done to characterize tau phosphorylation sites and map them to their kinases [30–35]. Proline-directed phosphorylation sites were decreased in *MAPT* V337M neurons, many of which serve as priming sites for additional sites of decreased tau phosphorylation (Figure S4B). Leveraging our global view of phosphorylation changes in *MAPT* V337M neurons, we predicted which kinases may have changes in activity based on known kinase-substrate relationships (Figure 3H). Kinases in the p38 MAPK pathway such as *MAP2K3* and *MAP2K6* were predicted to have increased activity in *MAPT* V337M neurons (Figure 3I). *MAPK11* and *MAPK14* targets had increased phosphorylation specifically in *MAPT* V337M neurons with tau knockdown, whereas *MAPK12* substrates had decreased phosphorylation specifically in *MAPT* WT neurons with tau knockdown. Known tau kinases with well-documented roles in tauopathy were also predicted to have differential activity, including *GSK3B*, *CDK5,* and *CDK5R1*. CDK5 and p38 MAPKs are both proline-directed kinases that are known to phosphorylate tau at several sites that had decreased phosphorylation in *MAPT* V337M neurons.

### CRISPR screens uncover regulators of tau phosphorylation in neurons

**Figure 4:**
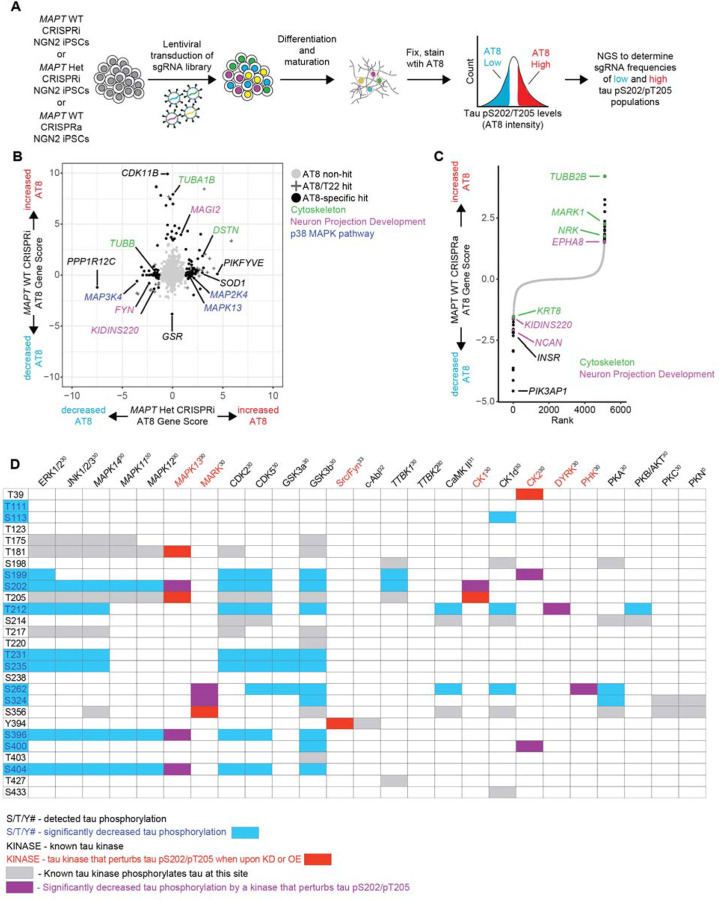
CRISPR screens elucidate regulators of tau phosphorylation in neurons. **(A)** Pooled genetic screening workflow for assessing changes in tau pS202/pT205 levels in neurons. *MAPT* WT CRISPRi NGN2 iPSCs, *MAPT* V337M heterozygous (*MAPT* Het) CRISPRi NGN2 iPSCs or *MAPT* WT CRISPRa NGN2 iPSCs were transduced by lentivirus with a pooled “druggable genome” sgRNA library targeting 2,318 genes enriched for kinases and phosphatases. iPSCs were differentiated into neurons and matured. After two weeks, neurons were fixed, stained with AT8 and sorted for high or low AT8 staining. The high AT8 and low AT8 samples were sequenced to determine which sgRNAs were enriched in either fraction. **(B)** Scatter plot comparing CRISPRi screens in *MAPT* Het neurons vs. *MAPT* WT neurons. AT8 non-hits are labeled with grey circles, AT8 hits that also modify tau levels (using the T22 antibody as a surrogate for total tau levels) are labeled with “+”, and AT8-specific hits are labeled with black circles. Top genotype-specific hits are labeled in black, and key pathways are labeled in green (cytoskeleton), purple (Neuron projection development) and blue (p38 MAPK pathway). **(C)** Rank plot showing the results of the CRISPRa screen in *MAPT* WT neurons. AT8 non-hits are labeled in grey and AT8 hits are labeled in black. Hits that are cytoskeleton-related genes are labeled in green and hits that are related to neuron projection development are labeled in purple. **(D)** Detected tau phosphosites are mapped to their known kinases. Tau phosphosites that were significantly different in either *MAPT* V337M homozygous (*MAPT* Hom) or *MAPT* Het neurons vs. *MAPT* WT are indicated with blue text/boxes. Kinases whose knockdown or overexpression perturb tau phosphorylation at S202/T205 are indicated by red text/boxes. Overlap between significant kinases and differential phosphorylations are indicated by purple boxes. Grey boxes indicate phosphorylations by known tau kinases that are not significantly differential or involved in tau pS202/T205. References for known tau kinase activity are indicated by the kinase name.

To directly test which kinases perturb tau phosphorylation in *MAPT* WT and *MAPT* V337M neurons, we employed CRISPRi and CRISPRa screens to test the effects of gene knockdown or overexpression on tau phosphorylation using the AT8 antibody, which detects the tau pS202/pT205 phosphoepitope (Figure 4A). We transduced iPSCs with a lentiviral sgRNA library targeting 2,325 genes encoding kinases, phosphatases and other proteins in the “druggable genome”[36]. Two weeks after differentiation, neurons were fixed and stained with AT8 and sorted based on AT8 signal. Next generation sequencing identified genes that causally regulate AT8 levels. We filtered out hits that also modified T22 levels in previously published work (Figure S5A) [37]. Cytoskeleton genes and genes involved in neuron projection development modified tau phosphorylation in both *MAPT* WT and *MAPT* V337M neurons (Figure 4A,B) without altering T22 levels (Figure S5A). Intriguingly, several kinases in the p38 MAPK pathway altered tau phosphorylation specifically in *MAPT* V337M neurons. Other kinases predicted to have differential activity that may have regulated tau phosphorylation in *MAPT* V337M neurons did not affect tau pS202/pT205 levels, including *CDK5*, *CDK5R1*, and *GSK3B* (Figure S5B). We mapped the detected tau phosphorylation sites in our neurons to their known kinases based on the literature, overlaying phosphorylation sites that were differential in *MAPT* V337M (blue) with kinases whose knockdown or overexpression modified tau phosphorylation at S202/T205 (red) (Figure 4D). The overlap between differential tau phosphorylation and kinases that regulate pS202/pT205 in neurons (purple) narrows the list down to a few candidate kinases. Overexpression of *MARK1*, a kinase that phosphorylates tau in the microtubule binding domain and regulates tau’s interaction with microtubules, caused increased tau phosphorylation in *MAPT* WT neurons. This is consistent with previous work showing that phosphorylation at S262, S324 and S356 affects phosphorylation sites distal from the microtubule binding domain, such as S202/T205 [38].

## Discussion

We have discovered that an FTD-causing variant of tau leads to tau hypophosphorylation and causes loss of tau function in regulating axonogenesis in differentiating neurons. These findings are surprising because disease-associated tau is typically associated with increased tau phosphorylation and toxic gain of function. Other groups have shown in mice or in primary neurons that reducing tau can have varying effects on axonogenesis. Acute tau ablation in mouse neurons *in vitro* prevents axonogenesis by inhibiting polarization [39, 40] and tau knockout in primary hippocampal neurons reduces neurite outgrowth and branching [41]. Our work also emphasized the importance of having iPSCs from multiple individuals and multiple clones paired with appropriate controls like tau knockdown and knockout.

There was substantial overlap between our RNA-seq findings and a recent paper using *MAPT* V337M neurons in an organoid model [9]. The other transcriptomic and proteomic signatures point to multiple layers of regulation around axonogenesis that are likely also in response to changes induced by *MAPT* V337M or tau loss.

The normal function of tau is unclear and has been debated for many years. This is in large part due to the many conflicting studies, both in a physiological and pathogenic context. Given the earlier results in showing the importance of tau for axonogenesis, it was expected that knocking out tau in mice would be lethal and that tau would be essential for neurodevelopment. Early mouse studies showed that tau knockout was surprisingly well tolerated [42]. There were no obvious defects in polarization or gross morphology, but microtubules in small caliber axons were destabilized. *Map1a* was upregulated in tau knockout mice, suggesting that the mice were compensating for tau loss. This could explain the difference in phenotypes as compared to the acute depletion of tau with ASOs. Knocking out tau and *Map1b,* another microtubule-associated protein, leads to much more severe phenotypes than either knockout individually [43]. Dawson et al. disputed the findings of Harada et al. due to poor WT data [44]. In their work, they found that indeed tau knockout did cause a delay in neurite outgrowth and axonogenesis.

Biswas and Kalil showed that tau knockout neurons had altered microtubule dynamics in growth cones, resulting in a change in overall growth cone morphology [45]. Microtubules were less bundled, and microtubule polymerization directionality as measured by EB3 was more dispersed in tau knockout neurons. There also was a reduction in tyrosinated tubulin projecting into the filopodia of the peripheral domain. Another paper showed that tau knockout increased Fyn mobility in dendrites and lowered Fyn localization in dendrites and spines [46]. Intriguingly, expressing P301L tau had the opposite effect and anchored or trapped Fyn in dendritic spines.

Many motor and behavioral phenotypes have been observed in tau knockout mice. Tau knockout mice or mice with acute tau reduction with antisense oligonucleotides have consistently shown resistance to seizures [21, 47–50]. Another consistent theme is that there are often behavioral and learning changes in tau knockout mice, including hyperactivity, fear conditioning, and memory [51–56]. There is more controversy over the effect of tau knockout on motor function. Some groups report motor deficits in tau knockout mice [51, 57, 58], while others claim there are no significant changes in tau knockout mice to motor function [47, 48, 56]. One group showed that tau is essential for long term depression in the hippocampus [59], while another showed that tau knockout only perturbs long term potentiation [56]. Tau phosphorylation has also been shown to be required for long term depression [60].

The loss-of-function phenotypes coincide with the onset of tau expression and axon extension, and the toxic gain-of-function phenotypes coincide with synaptogenesis and the onset of neuronal activity. These dual pathways provide further insight into how the *MAPT* V337M mutation dysregulate neurons in human disease. Tau loss of function would precede human disease onset by many decades, occurring during development and continuing through adulthood via perturbed synaptic plasticity. A study showed that mice with the *MAPT* P301L mutation show early cognitive changes before tau pathology is detectable [61]. A Parkinson’s disease GWAS study found that *MAPT* was a significant risk locus for Parkinson’s disease that is uncoupled from the age of onset [62]. Ye and colleagues proposed that tau may drive changes during development or early in life that then increase risk for Parkinson’s disease decades later [63]. Two studies have also identified cognitive differences between *MAPT* carriers and non-carrier siblings decades before expected disease onset [64, 65].

We acknowledge that there are limitations to our study. Our neurons under the conditions we used only express a single isoform of tau, the fetal isoform 0N3R. Understanding how different tau isoforms are regulated and how they contribute tau function in health and disease is an open question. Our findings would also be strengthened by additional work to directly measure the effects of V337M tau or tau loss on neurite outgrowth in our neurons. Additionally, it will be intriguing to understand how different disease variants of tau perturb neurons. Our data showing phosphorylation differences between WT tau, V337M tau and R406W tau joins a growing body of literature showing that different mutations have different effects on tau properties, including microtubule binding, microtubule polymerization, and fibril formation [66–72].

Taking all these data together, tau clearly has a role in axonogenesis during development. Given the strong link between neuronal excitability and neurodegenerative diseases like Alzheimer’s and Frontotemporal Dementia, a model of a vicious cycle where tau and neuronal excitability feedback on each other either from a tau-first insult (FTD) or neuronal excitability insult (AD) could provide a unifying mechanism explaining tauopathy pathogenesis.

## Materials and Methods

### Human iPSC culture and neuronal differentiation (Adapted from Tian et al. 2021)

Human iPSCs from the WTC11 background were cultured in StemFlex Medium (GIBCO/Thermo Fisher Scientific; Cat. No. A3349401). Human iPSCs from the GIH6C1 background were cultured in mTeSR Plus medium (StemCell Technologies; Cat. No. 100-0276). iPSCs were grown in plates or dishes coated with Growth Factor Reduced, Phenol Red-Free, LDEV-Free Matrigel Basement Membrane Matrix (Corning; Cat. No. 356231) diluted 1:100 in Knockout DMEM (GIBCO/Thermo Fisher Scientific; Cat. No. 10829-018). StemFlex Medium was replaced daily. When cells reached 80–90% confluency, cells were dissociated with StemPro Accutase Cell Dissociation Reagent (GIBCO/Thermo Fisher Scientific; Cat. No. A11105-01) at 37°C for 5 min, centrifuged at 200 g for 5 min, resuspended in StemFlex Medium supplemented with 10 nM Y-27632 dihydrochloride ROCK inhibitor (Tocris; Cat. No. 125410) and placed onto Matrigel-coated plates or dishes. Studies at UCSF with human iPSCs were approved by the Human Gamete, Embryo, and Stem Cell Research (GESCR) Committee.

For individual gene knockdown in CRISPRi iPSCs, sgRNAs were introduced into iPSCs via lentiviral delivery. Cells were selected by 1 μg/ml puromycin for 2–4 days and recovered for 2–4 days. Phenotypes were evaluated 5–7 days after infection.

The WTC11 CRISPRi iPSC lines were previously engineered to express mNGN2 under a doxycycline-inducible system in the AAVS1 safe harbor locus. The GIH6C1 iPSC lines were engineered in this work to express Ngn2 under a doxycycline-inducible promoter in the AAVS1 safe harbor locus. For their neuronal differentiation, we followed our previously described protocol [73]. Briefly, iPSCs were pre-differentiated in matrigel-coated plates or dishes in N2 Pre-Differentiation Medium containing the following: Knockout DMEM/F12 (GIBCO/Thermo Fisher Scientific; Cat. No. 12660-012) as the base, 1X MEM Non-Essential Amino Avids (GIBCO/Thermo Fisher Scientific; Cat. No. 17502-048), 10 ng/mL NT-3 (PeproTech; Cat. No. 450-03), 10 ng/mL BDNF (PeproTech; Cat. No. 450-02), 1μg/mL Mouse Laminin (Thermo Fisher Scientific; Cat. No. 23017-015), 10 nM ROCK inhibitor and 2μg/mL doxycycline to induce the expression of Ngn2. After three days, or Day 0, pre-differentiated cells were dissociated with accutase and plated into BioCoat Poly-D-Lysine-coated plates or dishes (Corning; assorted Cat. No.) in Classic N2 neuronal medium or BrainPhys Neuronal Medium. Classic N2 neuronal medium contained the following: half DMEM/F12 (GIBCO/Thermo Fisher Scientific; Cat. No. 11320-033) and half Neurobasal-A (GIBCO/Thermo Fisher Scientific; Cat. No. 10888-022) as the base, 1X MEM Non-Essential Amino Acids, 0.5X GlutaMAX Supplement (GIBCO/Thermo Fisher Scientific; Cat. No. 35050-061), 0.5X N2 Supplement, 0.5X B27 Supplement (GIBCO/Thermo Fisher Scientific; Cat. No. 17504-044), 10 ng/mL NT-3, 10 ng/mL BDNF and 1 μg/mL Mouse Laminin. BrainPhys Neuronal Medium was comprised of the following: BrainPhys Neuronal Medium (StemCell Technologies; Cat. No. 05791) as the base, 0.5x N2 Supplement, 0.5X B27 Supplement, 10 ng/mL NT-3, 10ng/mL BDNF, and 1 μg/mL Mouse Laminin. Neuronal medium was fully changed on day 3 post differentiation and then half-replaced on day 7 and weekly thereafter.

### GIHC1 iPSC cell line generation

GIH6C1 and GIH6C1∆1E11 were obtained from NeuraCell [23]. iPSCs were transfected with pC13N-dCas9-BFP-KRAB and TALENS targeting the human CLYBL intragenic safe harbor locus (between exons 2 and 3) (pZT-C13-R1 and pZT-C13-L1, Addgene #62196, #62197) using DNA In-Stem (VitaScientific). At the same time, the iPSCs were also transfected with E42 pUCM-TO-mNGN2 and TALENS targeting the human AAVS1 intragenic safe harbor locus (PLASMID IDs). After two weeks, BFP-positive iPSCs (CRISPRi+/mNGN2-), mCherry-positive iPSCs (CRISPRi-/mNGN2+) and BFP/mCherry-positive iPSCs (CRISPRi+/mNGN2+) were isolated via FACS sorting. Cells were plated sparsely in a 10 cm dish (5,000–10,000 per dish) and allowed to grow up until they formed large colonies. Homogenous BFP+/mCherry+ colonies were picked with a pipette tip and placed into a 24 well plate for expansion and characterization. Cre mRNA was then transfected into the iPSCs to remove the selection marker and mCherry. Cells were sorted for mCherry negativity, and then mCherry negative colonies were picked and genotyped.

### Western blots

Neurons were washed 3 times with ice-cold PBS. Ice-cold RIPA with protease and phosphatase inhibitors was added to cells. Lysates were incubated on ice for 2 minutes and then scraped down. Lysates were centrifuged at 12500xg for 10 minutes at 4 °C. The supernatants were collected, and the concentrations were measured with the BCA assay (Thermo Fisher Scientific; Cat No. 23225). 10–20 μg protein were loaded onto 4–12% Bis-Tris polyacrylamide gel (Thermo Fisher Scientific; Cat No. NP0336BOX) Nitrocellulose (BioRad, Cat. No. 1620146) or PVDF membranes were used to transfer the protein in a BioRad Transblot for 11 minutes at 25 V, 25 A. Membranes were then blocked for 1 hour with Licor Intercept blocking buffer (Licor, Cat. No. 927-60001) at room temperature. Primary antibody was added in Licor Intercept block overnight at 4 °C. Blots were washed 3 times for 5 minutes with TBST at room temperature. Secondary antibodies were added in Licor Intercept block for 1 hour at room temperature. Blots were washed 3 times for 5 minutes with TBST at room temperature and imaged on a Licor Odyssey. Immunoblots were quantified by intensity using ImageStudio (Licor).

### Bulk RNA sequencing sample preparation

RNA was harvested from day 7, day 14 and day 28 post differentiation neurons using a Zymo microprep kit (Zymo Research, Cat No. R2062). The library was prepared by first depleting ribosomal RNA (New England BioLabs, Cat No. E7405L). cDNA synthesis was then performed on all remaining RNAs (New England BioLabs, Cat. No. E7765S). Sequencing was performed at the Chan Zuckerberg Biohub and the UCSF Center for Advanced Techonlogy.

### ATAC-seq sample preparation

Cells were treated with Tn5 transposase to tag and cleave open chromatin with PCR adapters. Tagged sequences were purified, amplified and sequenced by high throughput sequencing.

### Proteomics sample preparation

Briefly, neurons were scraped off 15 cm dishes at day 7 of differentiation and flash frozen in liquid nitrogen. Cell pellet was lysed by adding 1 ml of 6 M GnHCl, 100mM Tris pH 8 and boiling at 95 C for 5 minutes two times with 5 min rest in between. DNA was sheared three times via probe sonication at 20% amplitude for 10 s., followed by 10 s of rest. Following sonication, samples were allowed to solubilize on ice for 20 mins before clearing cell debris by centrifugation at 16,000 x g for 10 mins and determining protein concentration was using Protein Thermo Scientific 660 assay. Enough lysate for 1 mg of protein was aliquoted and Tris 2-carboxyethyl phosphine (TCEP) and chloroacetamide (CAA) were added to each sample to a final concentration of 40 mM and 10 mM respecitively, before incubating for 10 min at 45 C with shaking. Guanidine was then diluted at least 1:5 with 100 mM Tris pH 8. Trypsin and LysC (Promega) were added at a 1:100 (enzyme:protein w:w) ratio (total protease:protein ratio of 1:50) and digested overnight at 37°C with shaking. Following digestion, 10% trifluoroacetic acid (TFA) was added to each sample to a final pH ~2. Samples were desalted under vacuum using Sep Pak tC18 cartridges (Waters). Each cartridge was activated with 1 mL 80% acetonitrile (ACN)/0.1% TFA, then equilibrated with 3 × 1 mL of 0.1% TFA. Cartridges were then washed with 4 × 1 mL of 0.1% TFA, and samples were eluted with 0.8 mL 50% ACN/0.25% formic acid (FA). 20 μg of each sample was kept for protein abundance measurements, and the remainder was used for phosphopeptide enrichment. Samples were dried by vacuum centrifugation.

### Phosphopeptide enrichment

For phosphopeptide enrichment of samples for phosphoproteomics, IMAC beads (Fe-IMAC from Cube Biotech) were prepared by washing 3x with washing buffer (0.1% TFA, 80% ACN). Dry, digested peptide samples were resuspended in washing buffer and incubated for 15 mins at 37 C with shaking. Peptides were enriched for phosphorylated peptides using a King Fisher Flex (KFF). A more detailed KFF protocol can be provided. Briefly, after resuspension peptides were mixed with beads and bound peptides were washed three times with wash buffer before being eluted from beads using 50% ACN, 2.5 % NH4OH solution. Enriched phosphorylated peptide samples were acidified using 75% ACN, 10% FA (at a ratio of 5:3 elution buffer:acid buffer), and filtered by centrifugation through NEST tips.

### Mass spectrometry data acquisition

Digested samples were analyzed on an Orbitrap Exploris 480 mass spectrometry system (Thermo Fisher Scientific) equipped with either an Easy nLC 1200 or Neo Vanquish ultra-high pressure liquid chromatography system (Thermo Fisher Scientific) interfaced via a Nanospray Flex source. Separation was performed using a 15 cm long PepSep column with a 150 um inner diameter packed with 1.5um Reprosil C18 particles. Mobile phase A consisted of 0.1% FA, and mobile phase B consisted of 0.1% FA/80% ACN. Abundance samples were separated by an organic gradient from 4% to 30% mobile phase B over 62 minutes followed by an increase to 45% B over 10 minutes, then held at 90% B for 8 minutes at a flow rate of 600 nL/minute. Phosphoproteomics samples were separated by an organic gradient from 2% to 25% mobile phase B over 62 minutes followed by an increase to 40% B over 10 minutes, then held at 95% B for 8 minutes at a flow rate of 600 nL/minute. To expand the spectral library, two samples from each set of biological replicates was acquired in a data dependent manner. Data dependent analysis (DDA) was performed by acquiring a full scan over a m/z range of 350–1100 in the Orbitrap at 60,000 resolving power (@200 m/z) with a normalized AGC target of 300%, an RF lens setting of 40%, and a maximum ion injection time of “Auto”. Dynamic exclusion was set to 45 seconds, with a 10 ppm exclusion width setting. Peptides with charge states 2–6 were selected for MS/MS interrogation using higher energy collisional dissociation (HCD), with 20 MS/MS scans per cycle. MS/MS scans were analyzed in the Orbitrap using isolation width of 1.6 m/z, normalized HCD collision energy of 30%, normalized AGC of 200% at a resolving power of 15,000 with a 22 ms maximum ion injection time. Similar settings were used for data dependent analysis of phosphopeptide-enriched and abundance samples. Data-independent analysis (DIA) was performed on all samples. An MS scan at 60,000 resolving power over a scan range of 350–1100 m/z, a normalized AGC target of 300%, an RF lens setting of 40%, and the maximum injection time set to “Auto”, followed by DIA scans using 20 m/z isolation windows over 350–1100 m/z with a 2 m/z overlap at a normalized HCD collision energy of 30%.

### Antibodies used in this study

cJun (CST, #9165)

p-cJun (CST, #91952)

Tau13 (Santa Cruz Biotechnology, sc-21796)

AT8 (Invitrogen, MN1020)

AT100 (Invitrogen, MN1060)

AT180 (Invitrogen, MN1040)

Tau pT217 (Invitrogen, 44–744)

Tau pS396 (Invitrogen, 44–752G)

GAPDH (Santa Cruz Biotechnology, sc-47724)

β-Actin (CST, #4967)

### Molecular Cloning:

Overexpression constructs were generated using our previously described PSAP expression vector [74] as a backbone. This vector expressed PSAP fused to a c-terminal mScarlett. We cloned emGFP-BRD2 into this vector (deleting PSAP-mScarlett) and then used XhoI and AgeI restriction enzyme sites to clone in 0N3R tau. We then cloned a gene block for ORF-BamHI-(GS)4-exFlag-T2A-mApple into the vector using the AgeI and EcoRI sites [37]. We then mutated WT 0N3R tau to V337M and R406W to generate the final overexpression constructs.

### CRISPR screening:

45 million iPSCs were infected with lentivirus encoding for the H1 sublibrary (Horlbeck et al Elife) at an MOI of ~0.3 and selected with 1ug/mL puromycin until 100% BFP positive. Lentivirus preparation was performed as described (https://dx.doi.org/10.17504/protocols.io.8dfhs3n, [37], [73]). For CRISPRa screens, TMP was added at a final concentration of 50uM for all cultures after selection. Cells were then differentiated and cultured as previously described (dx.doi.org/10.17504/protocols.io.bcrjiv4n, [73]). Upon differentiation, pre-differentiated cells were plated on three 15cm PDL-coated dishes at a density of 15 million cells per plate. Neurons were then matured for two weeks. At two weeks of age, neurons were lifted with papain and zinc fixed as previously described [37]. On the day of sorting, preparation for FACS was performed as described [37] using the AT8 antibody (Thermo MN1020) at a concentration of 1:200. After sorting, cells were pelleted at 200xg for 20 minutes, the supernatant was removed and the pellet was frozen at −20. Genomic DNA was extracted with the NucleoSpin Blood L kit. sgRNA cassettes were amplified, pooled, and sequenced as described [73]. CRISPR screens were analyzed using MAGeCK-iNC as previously described [73]. Briefly, raw sequencing reads were cropped and aligned using custom scripts that are publicly available (https://kampmannlab.ucsf.edu/resources). Raw phenotype scores and p-values were calculated for target genes and negative control genes using a Mann-Whitney U-test.

## Data Analysis

### RNA-seq analysis

Sequencing data was aligned to the human reference genome hg38. Rbowtie2 was used to align and count the number of transcripts from aligned reads. Differentially expressed genes were determined using DEseq2.

### ATAC-seq analysis

Sequencing data was aligned to the human reference genome hg38 using Rbowtie2. Peak calling was performed with MACS2. Differential ATACseq was performed using DEseq2, and motif analysis was performed with the motifDB and motifmatchr packages. Differential motif analysis was performed with the chromVar package.

### Gene set enrichment analysis

Enrichr was used to perform gene set enrichment analysis on RNA-seq, proteomics and phosphoproteomics datasets [75].

### Proteomics and Phosphoproteomics Analysis

Raw files were searched using the directDIA+ feature in Spectronaut, with DDA files provided as supplementary search files against a full human proteome from Uniprot (reviewed entries only, isoforms included). Phosphosites were extracted from the PTMsites output table from Spectronaut, and collapsed using the Tukey’s median polish functionality of MSstats in R.

## Supplementary Material

Supplement 1

Supplement 2

Supplement 3

Supplement 4

Supplement 5

Supplement 6
