# Supplementary material for: Multi-omic phenotyping of iPSC-derived neurons harboring the MAPT V337M mutation reveals tau hypophosphorylation and perturbed axon morphology pathways
Source: bioRxiv. 2025 Sep 9:2024.06.04.597496. Originally published 2024 Jun 6. Preprint. [Version 2] doi: 10.1101/2024.06.04.597496 (PMC11185762; doi:10.1101/2024.06.04.597496)
Supplement: Supplement 6 [file NIHPP2024.06.04.597496v2-supplement-6.pdf]

bioRxiv preprint doi: https://doi.org/10.1101/2024.06.04.597496; this version posted September 9, 2025. The copyright holder for this preprint (which was not certified by peer review) is the author/funder, who has granted bioRxiv a license to display the preprint in perpetuity. It is made available under aCC-BY 4.0 International license.

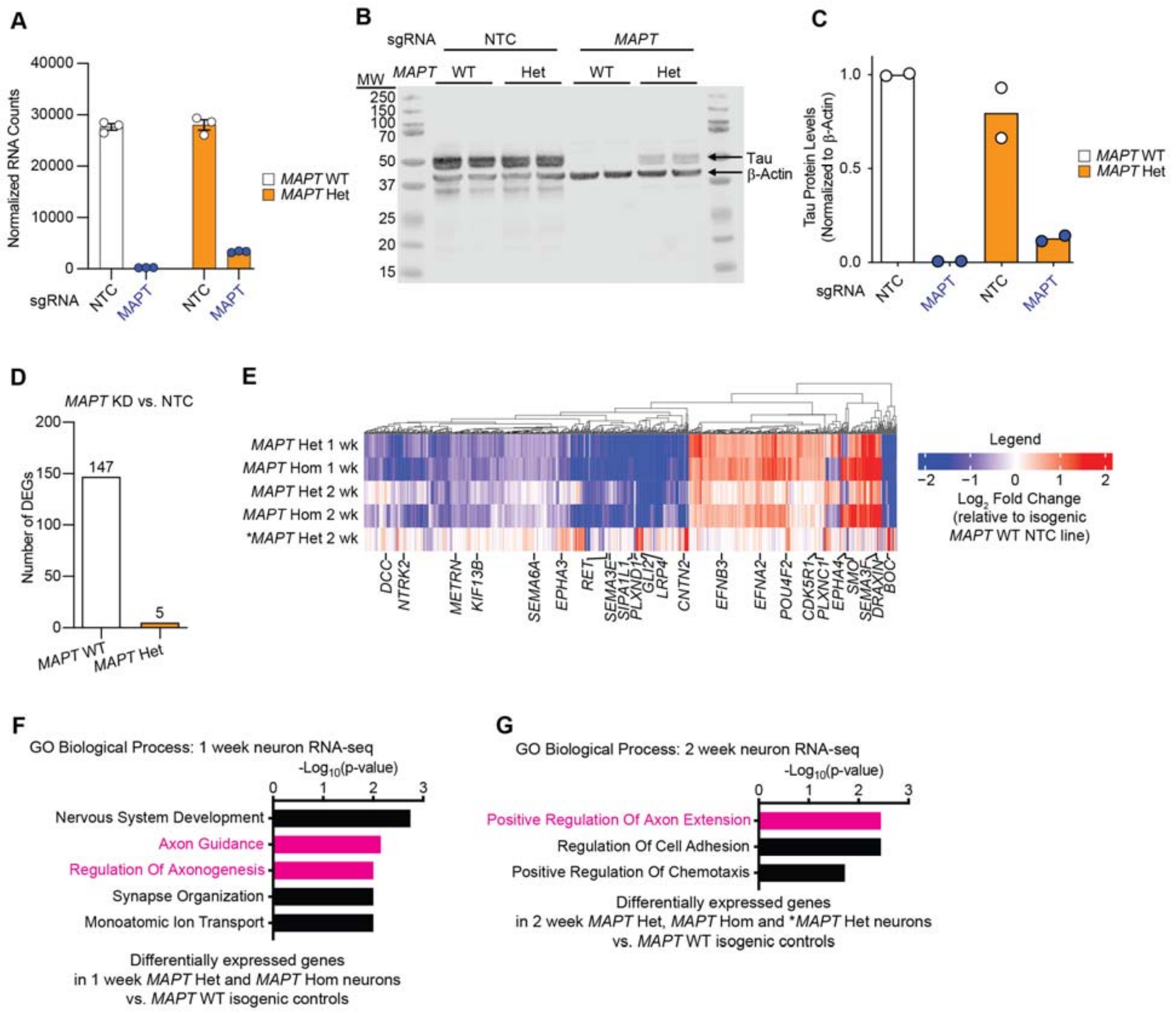

Figure S1: V337M tau and tau knockdown perturb gene expression of axonogenesis-related genes. (A) Normalized RNA counts of *MAPT* from the RNA-seq experiment described in Figure 1B showing tau knockdown in *MAPT* WT and *MAPT* Het neurons. **(B)** Western blot measuring tau knockdown in *MAPT* WT and *MAPT* Het neurons. Two replicates (individual wells) of neurons were harvested after two weeks of differentiation. **(C)** Quantification of the western blot in (B). **(D)** Bar plot showing the number of differentially expressed genes due to *MAPT* KD in either *MAPT* WT or *MAPT* Het neurons. **(E)** Heatmap of RNA-seq from *MAPT* Het, *MAPT* Hom and **MAPT* Het neurons vs. isogenic controls at 1 week or 2 weeks of differentiation.

bioRxiv preprint doi: https://doi.org/10.1101/2024.06.04.597496; this version posted September 9, 2025. The copyright holder for this preprint (which was not certified by peer review) is the author/funder, who has granted bioRxiv a license to display the preprint in perpetuity. It is made available under aCC-BY 4.0 International license.

Differentially expressed genes related to axon guidance or axonogenesis are labeled. **(F)** GO term enrichment analysis of one-week neurons from the RNA-seq experiment in (A). Genes that are differentially expressed in both *MAPT* Het and *MAPT* Hom vs. *MAPT* WT were analyzed with Enrichr, and top terms with minimal overlap were plotted. Pathways related to axonogenesis and neuron morphology are colored magenta. (G) GO term enrichment analysis of two-week old neurons from the RNA-seq experiment in (A). Genes that are differentially expressed in both *MAPT* Het, *MAPT* Hom and **MAPT* Het vs. their isogenic *MAPT* WT controls were analyzed with Enrichr, and top terms with minimal overlap were plotted. Pathways related to axonogenesis and neuron morphology are colored magenta.

bioRxiv preprint doi: https://doi.org/10.1101/2024.06.04.597496; this version posted September 9, 2025. The copyright holder for this preprint (which was not certified by peer review) is the author/funder, who has granted bioRxiv a license to display the preprint in perpetuity. It is made available under aCC-BY 4.0 International license.

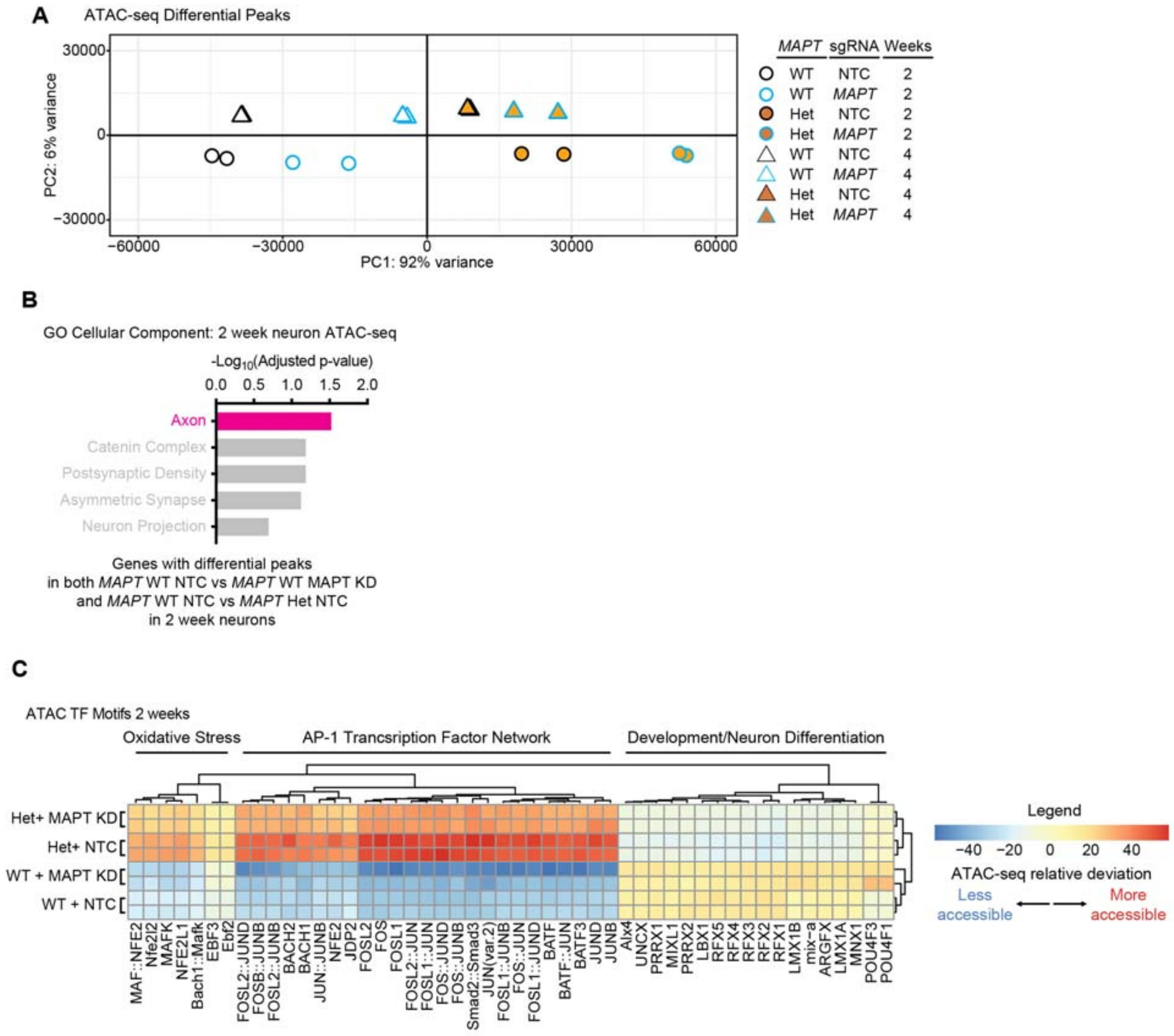

Figure S2: V337M tau and tau knockdown perturb chromatin accessibility of AP-1 transcription factor network motifs. (A) PCA plot of ATAC-seq differential peaks at 2 and 4 weeks of differentiation. Two replicates (individual wells) of neurons were harvested at each timepoint. **(B)** GO term enrichment analysis using Cellular Component on genes in 2-week neurons with differential ATAC-seq peaks in both *MAPT* WT *MAPT* KD and *MAPT* Het NTC vs. *MAPT* WT NTC. Non-significant terms are labeled in grey. **(C)** Heatmap showing the relative deviation of transcription factor motifs with significantly different accessibility in *MAPT*

bioRxiv preprint doi: https://doi.org/10.1101/2024.06.04.597496; this version posted September 9, 2025. The copyright holder for this preprint (which was not certified by peer review) is the author/funder, who has granted bioRxiv a license to display the preprint in perpetuity. It is made available under aCC-BY 4.0 International license.

WT and *MAPT* Het neurons +/- tau knockdown. Two replicates (individual wells) of neurons were harvested at two weeks of differentiation. GO term enrichment analysis was used on clusters of transcription factors to categorize clusters.

bioRxiv preprint doi: https://doi.org/10.1101/2024.06.04.597496; this version posted September 9, 2025. The copyright holder for this preprint (which was not certified by peer review) is the author/funder, who has granted bioRxiv a license to display the preprint in perpetuity. It is made available under aCC-BY 4.0 International license.

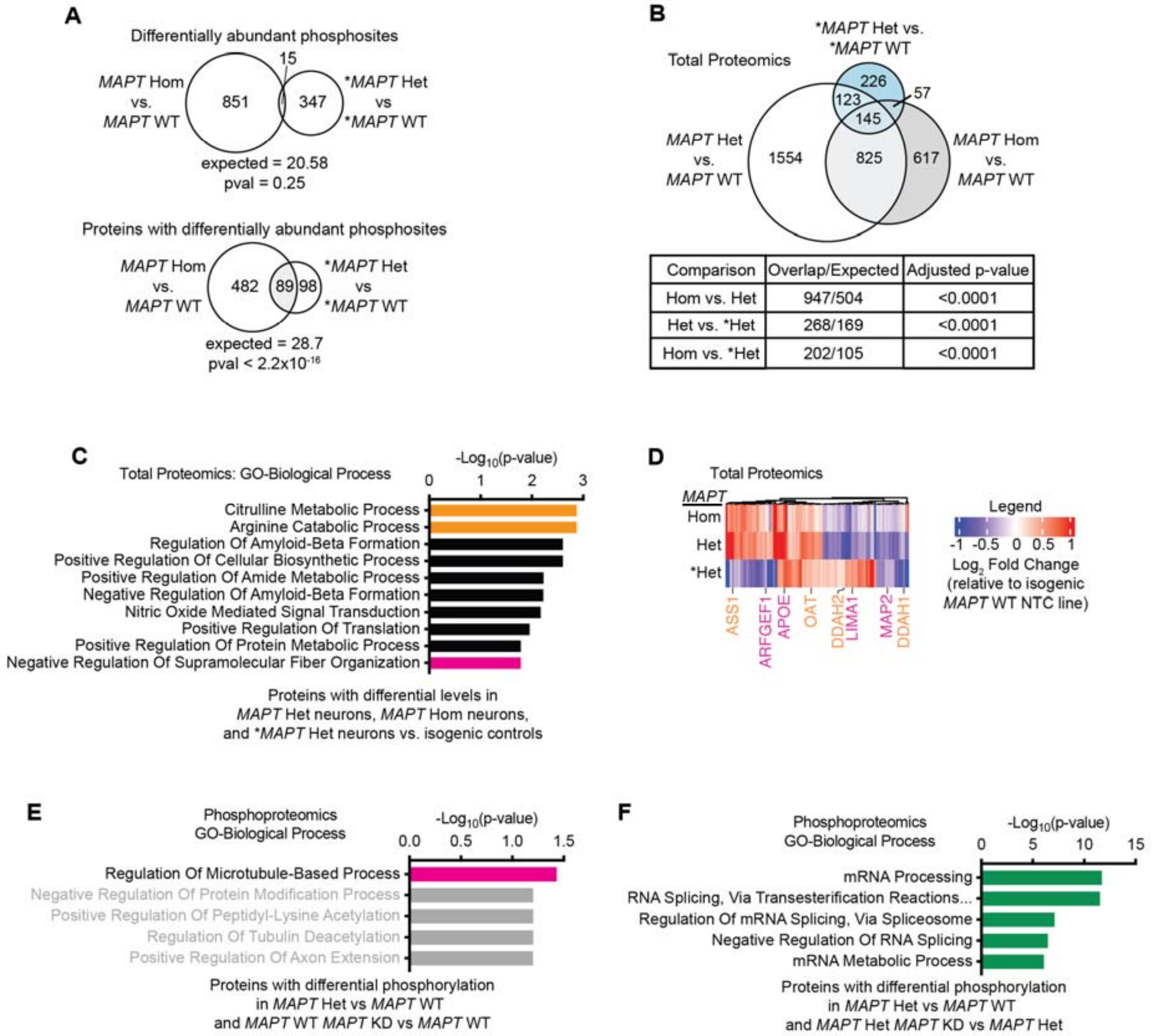

Figure S3: V337M tau and tau knockdown cause phosphorylation changes in axonogenesis and splicing proteins. (A) (*Top*) Overlap between differential phosphosites in neurons derived from iPSCs edited to introduce the homozygous *MAPT* V337M mutation (*MAPT* Hom)vs. isogenic controls (*MAPT* WT). Four replicates (independent 150mm dishes) of neurons for each genotype/sgRNA combination were harvested after one week of differentiation, and the phosphoproteome was measured using mass spectrometry. Significance was calculated using Fisher's Exact Test. (*Bottom*) Overlap between proteins with differential phosphorylation in both

bioRxiv preprint doi: https://doi.org/10.1101/2024.06.04.597496; this version posted September 9, 2025. The copyright holder for this preprint (which was not certified by peer review) is the author/funder, who has granted bioRxiv a license to display the preprint in perpetuity. It is made available under aCC-BY 4.0 International license.

datasets. Significance was calculated using Fisher's Exact Test. **(B)** Overlap between proteomic changes in *MAPT* Hom neurons, neurons derived from iPSCs edited to have the heterozygous *MAPT* V337M mutation (*MAPT* Het) and neurons derived from patient iPSCs with the heterozygous *MAPT* V337M mutation (**MAPT* Het) vs. isogenic controls (*MAPT* WT or **MAPT* WT). Four replicates (independent 150mm dishes) of neurons for each genotype/sgRNA combination were harvested after one week of differentiation, and the total proteome was measured using mass spectrometry. Significance was calculated using multiple t-tests adjusted with Šidák single-step correction. Significantly differential proteins in all three datasets were filtered to identify 145 conserved proteins. **(C)** GO term enrichment of the 145 proteins with differential abundance in *MAPT* Hom, *MAPT* Het and **MAPT* Het neurons compared to isogenic controls. Top terms with minimal overlap are shown. Term names are colored to match relevant gene names in the heatmap in (C). **(D)** Heatmap showing the Log_2 fold change of protein abundance for the 145 proteins with differential abundance in *MAPT* Hom, *MAPT* Het and **MAPT* Het neurons vs. isogenic *MAPT* WT neurons. Proteins within enriched GO terms are labeled and colored according to the shared pathways. **(E)** GO term analysis of phospoproteins with differential phosphorylation in *MAPT* Het NTC and *MAPT* WT *MAPT* KD vs. *MAPT* WT NTC. Non-significant terms are labeled by grey bars. Regulation of Microtubule-based process is labeled by a magenta bar due to its overlap with axon-related terms. **(F)** GO term analysis of phosphoproteins with differential phosphorylation in *MAPT* Het NTC vs. *MAPT* WT NTC and *MAPT* Het *MAPT* KD vs. *MAPT* Het NTC. Terms related to RNA processing and splicing are marked by green bars.

bioRxiv preprint doi: https://doi.org/10.1101/2024.06.04.597496; this version posted September 9, 2025. The copyright holder for this preprint (which was not certified by peer review) is the author/funder, who has granted bioRxiv a license to display the preprint in perpetuity. It is made available under aCC-BY 4.0 International license.

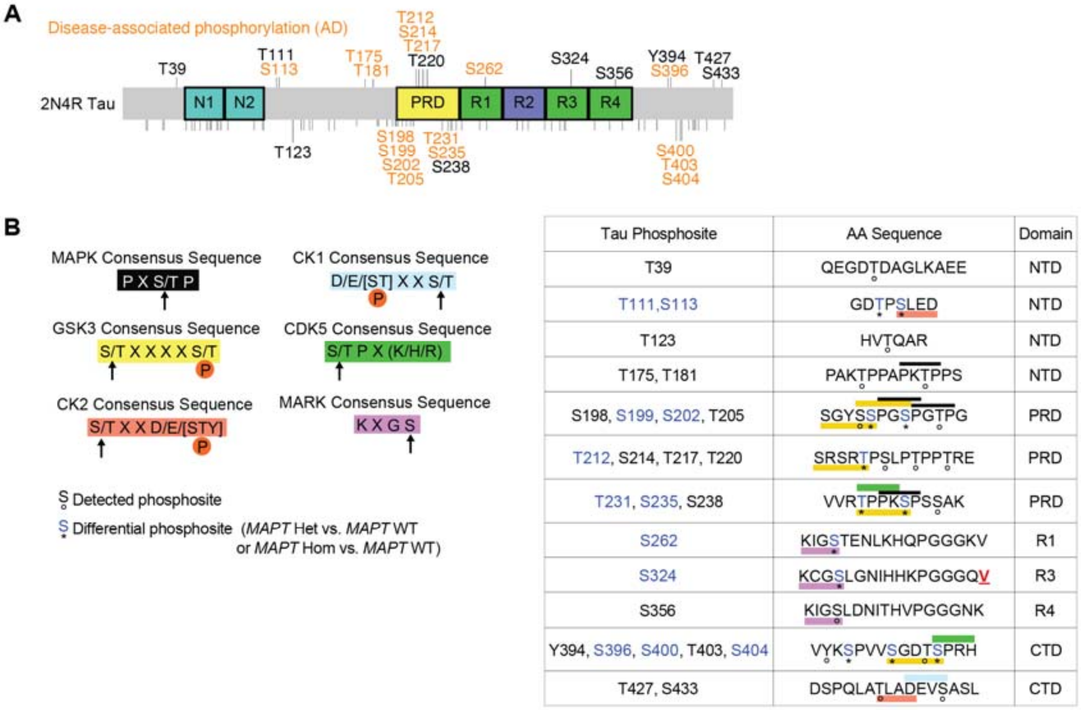

Figure S4: Neurons with V337M tau have decreased tau phosphorylation at disease-associated phosphorylation sites. (A) Protein domain map of 2N4R tau. Phosphosites detected in this study are labeled, with disease-associated phosphorylation sites from AD labeled in orange. Phosphosites not detected in this study are marked with a small black line and are unlabeled. Domain abbreviations are as follows: N-terminal inserts (N1,N2), proline rich domain (PRD), microtubule binding repeats (R1, R2, R3, R4). **(B)** Consensus sequences for tau kinases. Detected tau phosphosites are shown with their sequence context. Phosphorylation sites that are differential between either *MAPT* V337M heterozygous (*MAPT* Het) or *MAPT* V337M homozygous (*MAPT* Hom) are labeled blue with an asterisk, and detected phosphosites are labeled with an open circle. Kinase consensus sequences are annotated with colored boxes, with priming sites marked with a “P” in an orange circle. V337 is labeled with a bold/underlined red V. The domains abbreviated as follows: N-terminal projection domain (NTD), proline rich domain (PRD), Microtubule binding repeats (R1, R3, R4), C-terminal domain (CTD).

bioRxiv preprint doi: https://doi.org/10.1101/2024.06.04.597496; this version posted September 9, 2025. The copyright holder for this preprint (which was not certified by peer review) is the author/funder, who has granted bioRxiv a license to display the preprint in perpetuity. It is made available under aCC-BY 4.0 International license.

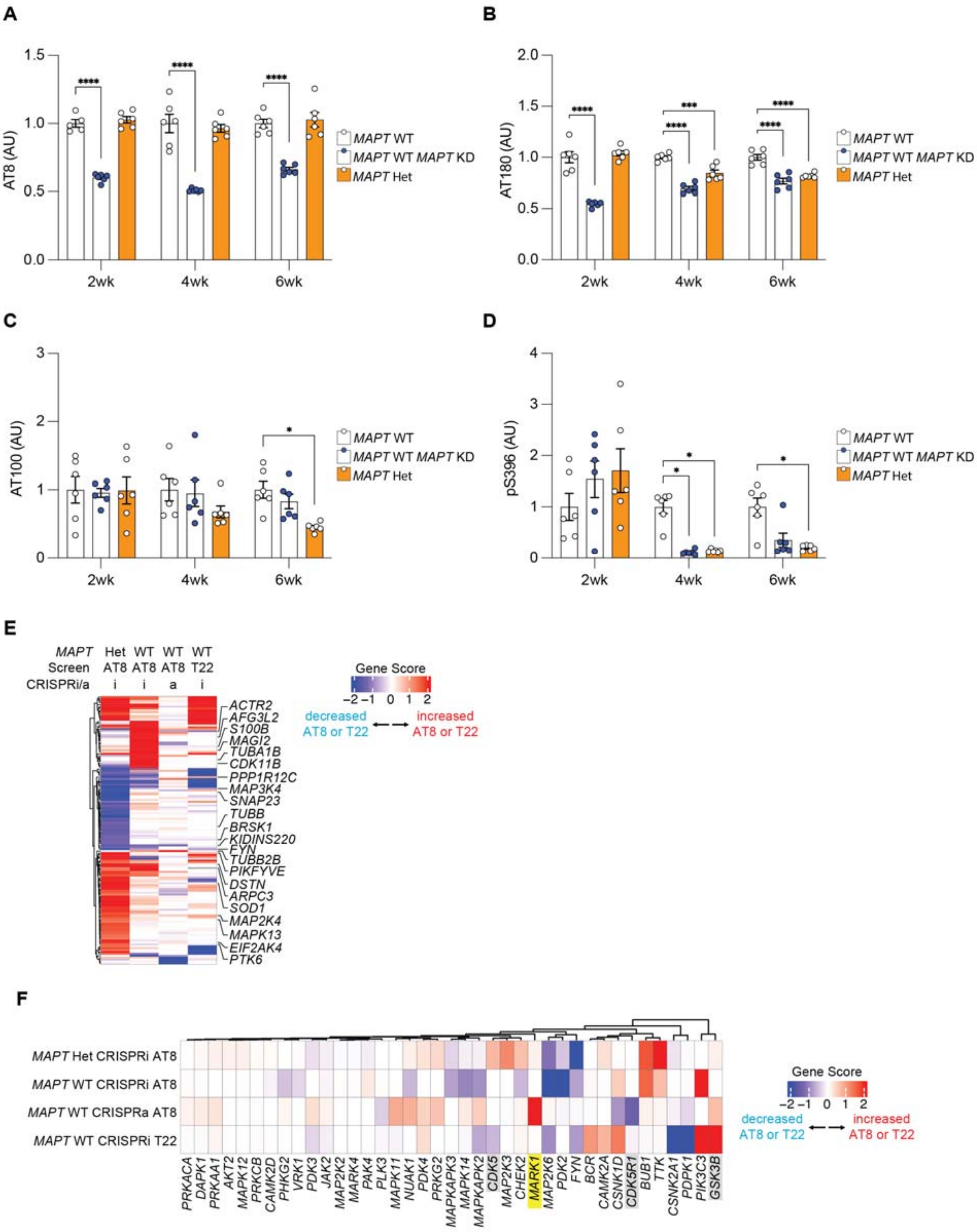

bioRxiv preprint doi: https://doi.org/10.1101/2024.06.04.597496; this version posted September 9, 2025. The copyright holder for this preprint (which was not certified by peer review) is the author/funder, who has granted bioRxiv a license to display the preprint in perpetuity. It is made available under aCC-BY 4.0 International license.

Figure S5: Functional genomics uncovers regulators of tau phosphorylation in *MAPT* WT and *MAPT* V337M neurons. (A-D) Bar plots showing the median intensity of AT8 (A), AT180 (B), AT100 (C) and pS396 (D) in 2-week *MAPT* WT, *MAPT* KD and *MAPT* Het neurons. AT8 was selected for CRISPR screening due to high reproducibility across timepoints and AT8 detection in both *MAPT* WT and *MAPT* Het neurons at 2 weeks of differentiaton. **(E)** Heatmap of hits from the CRISPRi and CRISPRa AT8 screens and the CRISPRi T22 screen. Many of the AT8 hits from the three screens do not modify T22 levels and are therefore unlikely to be due to modifying tau levels [37]. Genes related to cytoskeleton, neuron projection development or the p38 MAPK pathway are annotated. **(F)** Heatmap of AT8 and T22 screens with the kinases predicted to have differential activity in Figure 3I. Selected kinases predicted to have differential activity in *MAPT* V337M neurons with particular disease relevance that did not have a phenotype in the AT8 screens are highlighted with grey boxes. *MARK1* is annotated with a yellow box.

bioRxiv preprint doi: https://doi.org/10.1101/2024.06.04.597496; this version posted September 9, 2025. The copyright holder for this preprint (which was not certified by peer review) is the author/funder, who has granted bioRxiv a license to display the preprint in perpetuity. It is made available under aCC-BY 4.0 International license.

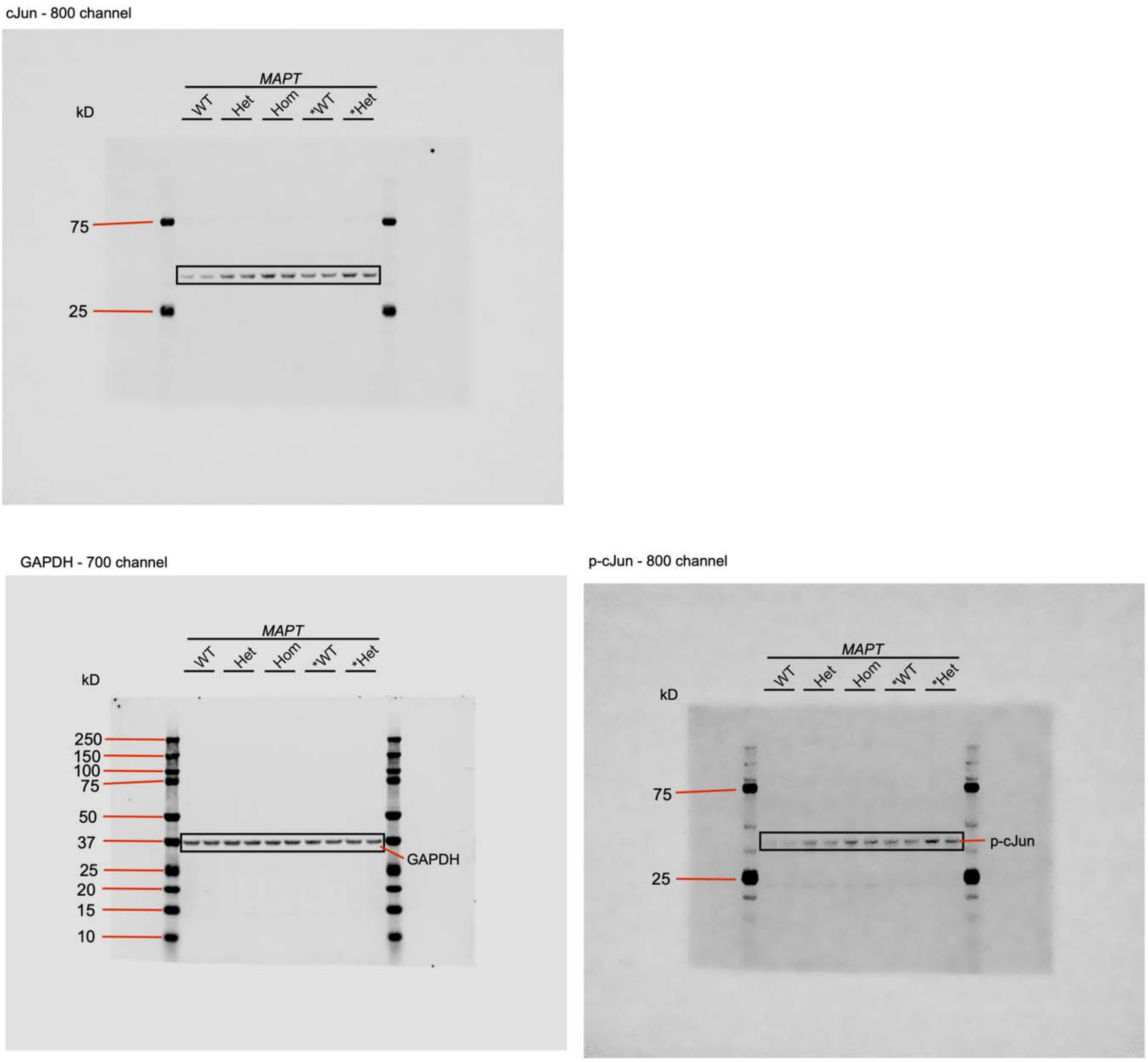

Source data for Fig. 1F

bioRxiv preprint doi: https://doi.org/10.1101/2024.06.04.597496; this version posted September 9, 2025. The copyright holder for this preprint (which was not certified by peer review) is the author/funder, who has granted bioRxiv a license to display the preprint in perpetuity. It is made available under aCC-BY 4.0 International license.

tau13

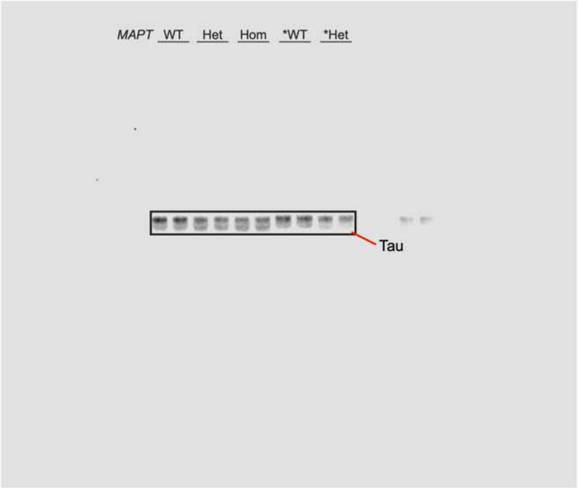

tau pS202/pT205 (AT8)

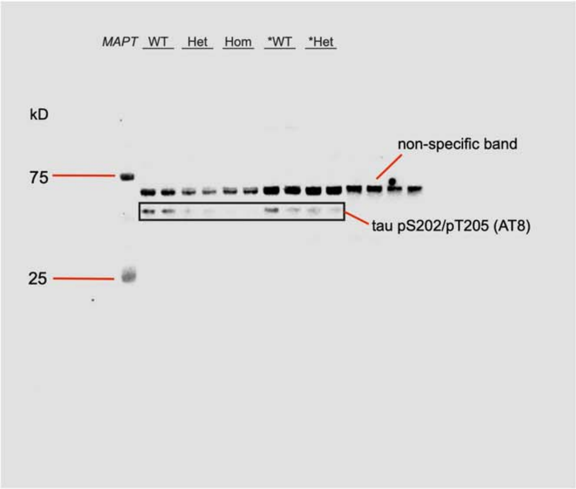

Actin

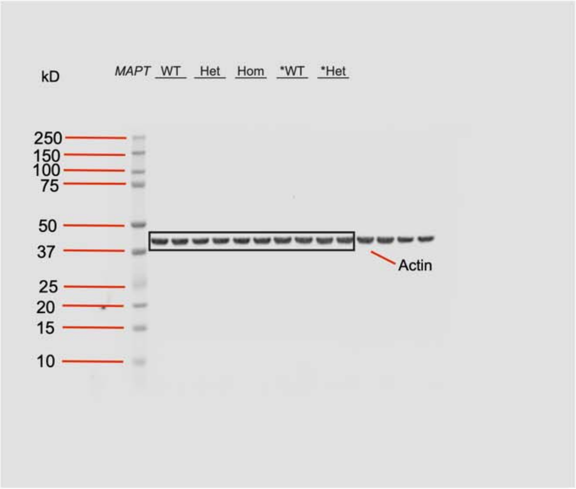

Source data for Fig. 3C

bioRxiv preprint doi: https://doi.org/10.1101/2024.06.04.597496; this version posted September 9, 2025. The copyright holder for this preprint (which was not certified by peer review) is the author/funder, who has granted bioRxiv a license to display the preprint in perpetuity. It is made available under aCC-BY 4.0 International license.

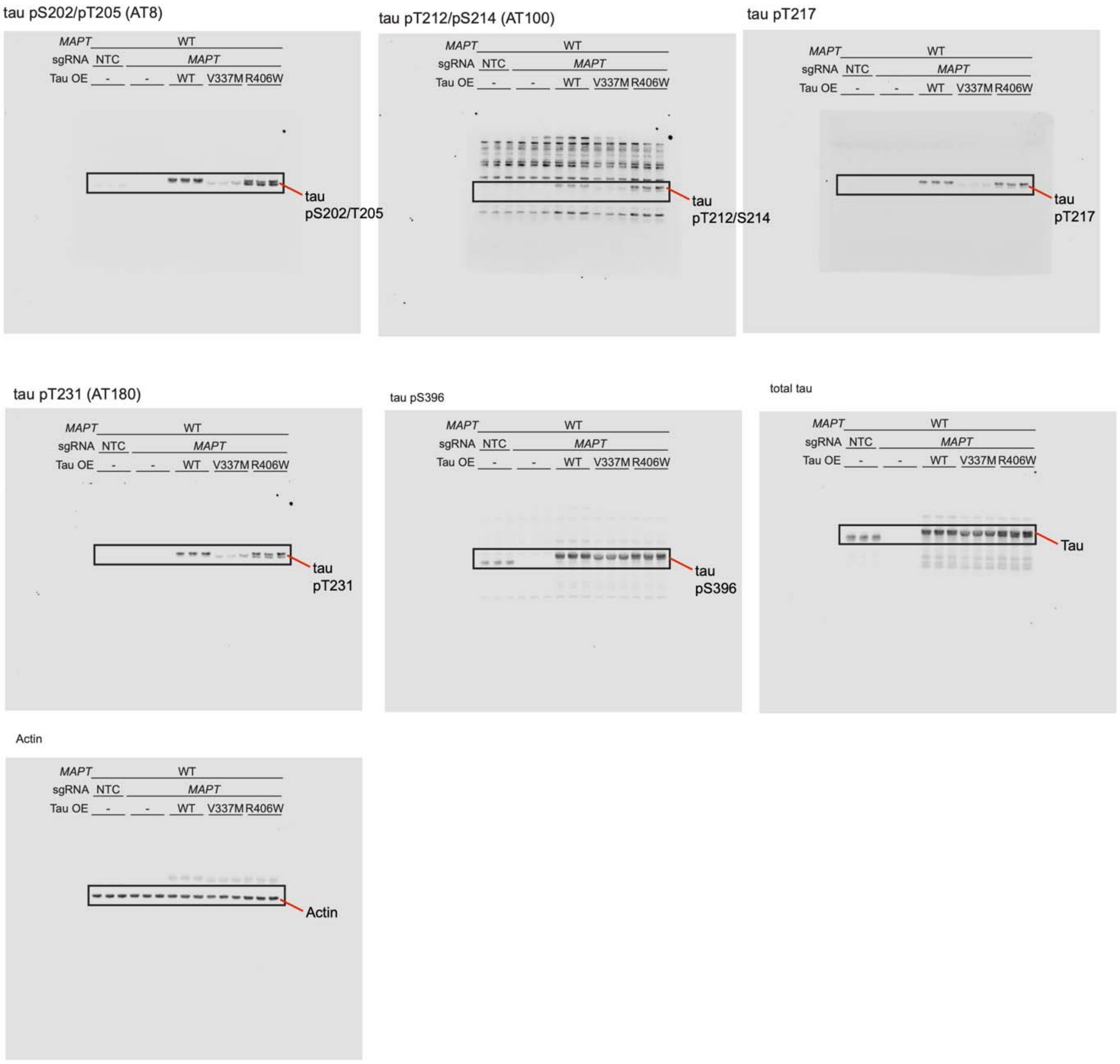

Source data for Fig. 3F
